# No need to invoke ‘unconscious attention' to explain the perceptual advantage of targets presented to the same eye as a preceding cue

**DOI:** 10.3389/fpsyg.2026.1840646

**Published:** 2026-09-23

**Authors:** Surya Gayet

**Affiliations:** Experimental Psychology, Helmholtz Institute, Utrecht University, Utrecht, Netherlands

**Keywords:** attention, binocular rivalry, flash suppression, stereopsis, unconscious processing

## Background

In their recent article “*Reflexive attention can be driven by monocular cues without conscious access to eye of origin information*” (Fuentes and Valle-Inclán 2026) report that visual targets presented to one eye are detected faster when presented to the same eye as a preceding monocular cue than when presented to the other eye (Figure 1). Because observers are typically unable to discriminate which of their two eyes is being stimulated (Blake and Cormack, 1979), the authors interpret this validity effect as evidence that unconscious eye-of-origin information can guide reflexive attention, thereby supporting a dissociation between attention and awareness. While the behavioral effect itself appears robust, it can be fully accounted for by a much simpler explanation: presenting a cue to one eye temporarily shifts sensory eye dominance, thus temporarily enhancing visual processing in the eye to which the cue was presented. The current design does not allow one to distinguish this well-established low-level sensory account from an attentional orienting explanation. Addressing such alternative accounts is important because, in this field of research, conclusions about unconscious processing cumulatively add to the corpus of evidence for processes that are believed to operate in the absence of awareness. The aim of the present article is therefore to clarify the inferential limits of the reported findings and to outline a more parsimonious account that does not require the involvement of unconscious attentional orienting.

**Figure 1:**
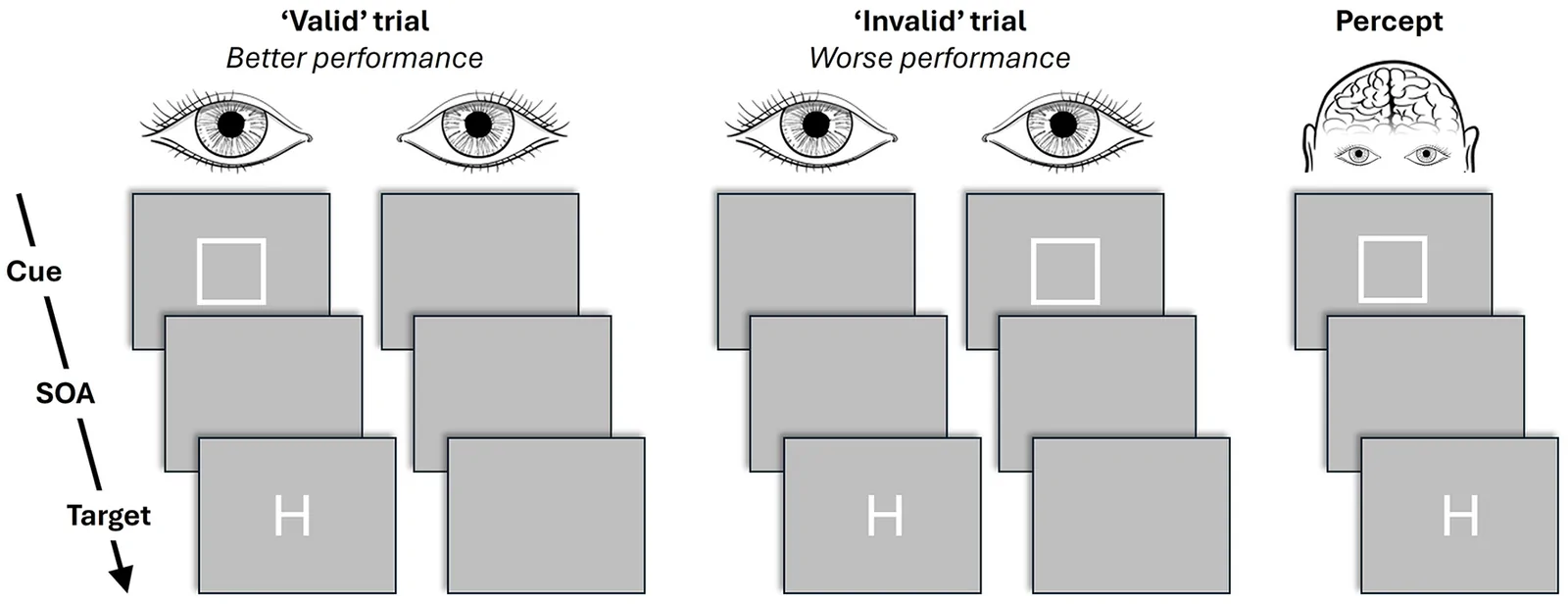
Schematic depiction of the experimental task by (Fuentes and Valle-Inclán 2026). Participants performed a speeded detection task on a target (the letter ‘H') following a cue. The cue and target were either presented to the same eye (‘valid' trial; **left**) or to different eyes (‘invalid' trial; **middle**). Participants viewed the images presented to the two eyes through a mirror stereoscope, fusing them into a single percept **(right)**, in which the cue and target stimuli all appeared at fixation. Theoretically, the valid and invalid trial types are perceptually indistinguishable, as observers have no access to eye-of-origin information (but see Sari et al., 2024). The time between cue and target onset (stimulus onset asynchrony; SOA) was varied.

In the original experiments, participants viewed stimuli through a mirror stereoscope. A brief monocular cue was presented to one eye, followed after a variable stimulus-onset asynchrony by a target letter (“H”), which participants detected as quickly as possible. Critically, cue validity was defined not in spatial terms, as in a classical Posner cueing paradigm (Posner, 1980), but in terms of eye of origin: trials were considered valid when cue and target were presented to the same eye, and invalid when they were presented to different eyes. Thus, the key behavioral finding was that targets were detected faster when they appeared in the same eye as the preceding cue than when they appeared in the other eye.

## Cue validity is fully confounded with sensory eye dominance

Presenting a monocular cue to one eye is known to transiently bias ocular dominance (e.g., Wolfe, 1984). Following brief monocular stimulation, the stimulated eye becomes perceptually dominant, while the other eye becomes perceptually recessive (Tsuchiya et al., 2006). Consequently, a subsequent target presented to the cued eye will be processed under conditions of higher sensory gain than a target presented to the other eye.

In the present experiments, “valid” trials are precisely those in which the target is presented to the same eye as the cue. “Invalid” trials are those in which the target appears in the opposite eye. Thus, cue validity is structurally confounded with dominant-vs.-recessive eye stimulation. If the cue enhances the sensory gain of the cued eye then valid targets should elicit faster responses than invalid targets, exactly as was found in the article by (Fuentes and Valle-Inclán 2026). The authors interpret this validity effect as evidence that eye-of-origin information, though inaccessible to awareness, drives reflexive attention. This validity effect, however, can be fully explained by processes occurring at the level of early sensory competition, without the need to invoke downstream attentional selection processes.

## Sensory gating vs. attention

It may be argued that the boundary between sensory competition and attention is not always sharply defined. If attention is defined broadly as the unequal allocation of processing resources, then a cue-induced shift in ocular dominance could itself be described as a shift of resources toward one eye's input. Following this broad conceptualization of attention, the present findings could be interpreted as showing that a monocular cue biases processing toward the stimulated eye, much as an exogenous cue biases processing toward a particular region of space in the classical Posner paradigm (Posner, 1980). Indeed, previous work shows that attention can be directed to stimulus content presented to one eye, thereby increasing its perceptual dominance during interocular competition (Ooi and He, 1999; Mitchell et al., 2004; Zhang et al., 2012; for a review, see Paffen and Alais, 2011). Moreover, spatial cueing effects can depend on whether cue and target are presented to the same or different eyes: Self and Roelfsema (2010) found that exogenous spatial cueing transferred across eyes at longer SOAs (150 and 400 ms), but was eye-specific at very short SOAs (50 ms). These findings show that attentional selection and interocular competition can interact; they can even be taken as evidence that (spatial) attention is affected by task differences (i.e., within vs. between eye cueing) that are presumably not accessible to consciousness. However, these findings do not imply that same-eye cueing benefits necessarily reflect attentional orienting. In the present case, the behavioral advantage to targets presented to the same eye as the cue can be explained more parsimoniously by early interocular competition: the cue transiently increases the dominance, or sensory gain, of the eye to which it was presented (Wolfe, 1984; Tsuchiya et al., 2006). This distinction matters because (Fuentes and Valle-Inclán 2026) explicitly frame their findings as evidence for reflexive attention driven by unconscious eye-of-origin information, and explicitly not merely as changes in the effective strength of one eye's input due to a cue-induced reversal of ocular dominance. Moreover, interocular competition can be understood as an early sensory gating mechanism, preceding later attentional amplification; perceptual dominance during rivalry has been linked to activity as early as the lateral geniculate nucleus (Wunderlich et al., 2005; Haynes et al., 2005), and rivalry-like alternations can even emerge in minimal systems consisting of mutually inhibitory units (Kogo et al., 2021). Thus, while attention and ocular dominance both alter sensory gain, they are typically considered distinct phenomena. Accordingly, the same-eye advantage reported by Fuentes and Valle-Inclán does not by itself establish that unconscious eye-of-origin information guided reflexive attention.

## Can the dynamics of the validity effect adjudicate between the two accounts?

Single flash suppression is a transient interocular effect. Suppression produced by a single brief flash typically peaks around 85–100 ms, with little to no measurable suppression beyond 200–300 ms (Tsuchiya et al., 2006). The temporal profile of the validity effect reported by Fuentes and Valle-Inclán is thus fully compatible with the time course of ocular dominance in flash suppression: it is absent at 0 ms, peaks at 100 ms, and dissipates from 200 ms onward.

This leaves unanswered, however, why Fuentes and Valle-Inclán observed no validity effect at an SOA of 50 ms, when ocular dominance effects should already be present (and near-peak). A likely explanation is that the cue caused an overall alerting effect, which helped target detection, and peaked at an SOA of 100 ms (thus reducing variance, and increasing statistical power to detect a validity effect). In line with this possibility, overall performance (collapsed across valid and invalid conditions) indeed peaked at 100 ms. Taken together, the dynamics of the validity effect are fully compatible with a transient, ocular-dominance induced account.

## The neutral condition does not solve the problem

Experiment 3 introduces a “neutral” condition where cues are presented binocularly, revealing a “pure benefit” pattern (valid RT < neutral ≈ invalid). Fuentes and Valle-Inclán take this as an argument against the ocular-dominance account, which would predict worse performance in the invalid than the neutral condition. While this is true, any attention-based explanation would similarly predict worse performance in the invalid compared to the neutral condition. The more general problem with the binocular control condition is that the cue has twice as much stimulus energy as in the other conditions, complicating a direct comparison of performances.

What the original study would need to support its claims, is a manipulation that orthogonally varies (1) sensory eye dominance and (2) cue validity. In other words, one would need to show a behavioral benefit in the cued eye compared to the uncued eye, using a cue that cannot trigger a switch in ocular dominance. It is not obvious that this can be achieved by refining the current design, because any monocular presentation is likely to alter interocular competition (whether manipulated through exogenous or endogenous cueing; e.g., Zhang et al., 2012; Ooi and He, 1999). More generally, one may wonder whether attention directed at an individual eye's input is even a meaningful concept. Thus, research on unconscious attention may benefit more from traditional approaches, whereby the cue is perceptually suppressed, and does not inherently drive early sensory gating of the valid target (e.g., Jiang et al., 2006; Rothkirch et al., 2012; Gayet et al., 2014; for reviews, see Mulckhuyse and Theeuwes, 2010; Prasad and Mishra, 2019).

## Is eye-of-origin information truly outside of conscious access?

Even if the eye dominance confound were somehow controlled for, a second issue remains: the claim that eye-of-origin information was inaccessible to awareness. Although often assumed, it is not accurate to state that human observers have no access to eye-of-origin information. Healthy observers can retrieve some eye-of-origin information for low-spatial frequency stimuli, and observers with stereo-blindness (about 5% of the population; Richards, 1970; Chopin et al., 2019) may even access eye-of-origin information for high spatial frequency information as well (Blake and Cormack, 1979). Moreover, in imperfect stereoscope setups, visual leakage between the two eyes' images may further increase access to eye-of-origin information (e.g., Sari et al., 2024). Thus, how sure can we be that participants had no conscious access to eye-of-origin information?

Fuentes and Valle-Inclán assessed access to eye-of-origin information in an awareness-screening block separate from the main task. However, offline visibility assessments can underestimate awareness and fail to capture trial-to-trial fluctuations in awareness (Pratte and Rouder, 2009; Lin and Murray, 2014; Mathewson et al., 2009; for discussion, see Stockart et al., 2025). Thus, even if the validity effect observed by Fuentes and Valle-Inclán (partially) reflected eye-specific attentional modulations, the method used to assess unawareness of eye-of-origin information would still be insufficient to support claims about its unconscious nature.

## Conclusion

The present commentary is not intended to argue against the possibility that attention and consciousness can be dissociated. This theoretically important dissociation has found substantial basis in empirical work, and has been widely discussed (Koch and Tsuchiya, 2007). Rather, the present commentary uses (Fuentes and Valle-Inclán 2026) as a concrete case illustrating the methodological care required when making claims about such dissociations. Research on unconscious processing, in particular, faces two main challenges. Firstly, conclusions about (cognitive or perceptual) processes that occur in the absence of conscious awareness are much more appealing to share, publish, and communicate, than the conclusion that these processes do not occur in the absence of awareness, thus inherently favoring the spread of research claiming unconscious processing. Secondly, once such evidence is out, it shapes the general belief about unconscious processing in a cumulative manner: every finding (whether spurious, or true) will lengthen the list of processes that are generally believed to operate in the absence of conscious awareness. In contrast, evidence to the contrary, or alternative low-level sensory accounts, may receive less visibility (and fail to shorten the list). Because of this inherent imbalance in the field, scientific rigor is of particular importance in the study of unconscious processing, and alternative accounts must be exhaustively ruled out before making claims about unconscious processing. For this reason, claims about unconscious attention require rigorous evidence, with particular focus on two separable points: first, that the observed behavioral enhancement reflects attentional selection rather than lower-level changes in sensory gating; and second, that the critical information (here: eye-of-origin) was genuinely unavailable to awareness. In the present case, neither requirement is fulfilled. The same-eye advantage can be explained more parsimoniously by transient, cue-induced shifts in ocular dominance, and the available awareness measure does not establish that eye-of-origin information was unavailable during the main task. Thus, while the findings discussed here do not challenge the broader possibility of unconscious attention, they also do not provide compelling evidence for it.
